# The effect of microbiome on social spacing in *Drosophila melanogaster* depends on genetic background and sex

**DOI:** 10.17912/micropub.biology.001270

**Published:** 2024-09-11

**Authors:** Yanira Jiménez-Padilla, Yen Chan, M. Sol Aletta, Marc-André Lachance, Anne F Simon

**Affiliations:** 1 Biology Department, University of Western Ontario, London, Ontario, Canada

## Abstract

The gut microbiome modulates many essential functions including metabolism, immunity, and behaviour. Specifically, within behaviour, social behaviours such as sociability, aggregation, mating preference, avoidance, oviposition, and aggression are known to be regulated in part by this host-microbiome relationship. Here, we show the microbiome's role in the determination of social spacing in a sex- and genotype-specific manner. Future work can be done on characterizing the microbiome in each of these fly strains to identify the species of microbes present as well as their abundance.

**Figure 1. Social space of flies axenic or not, for 4 different strains:**
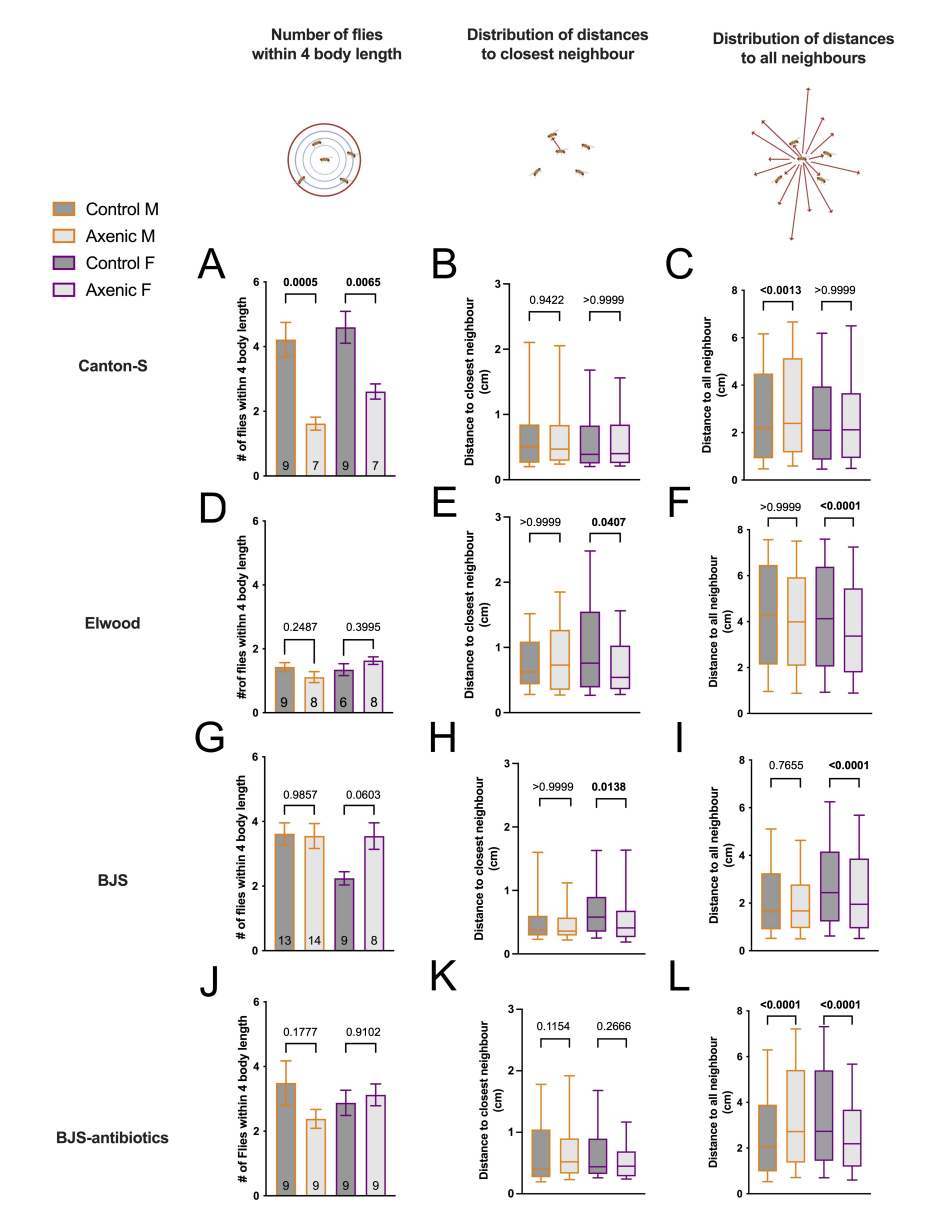
Canton-S **(A-C),** Elwood **(D-E),** BJS **(G-I)** and BJS treated with antibiotics **(J-L). A, D, G, J:** Social space measured as the average number of flies within 4 body length, +/- s.e.m., number of assays indicated in the columns, each with 12-17 flies of indicated sexes. **B, E, H, K:** Social space of the same assays measured as the distribution of distance to the closest neighbour, and **C, F, I, L** distribution of the distance to all neighbours, represented with box (median, and interquartile 25-75%) and whiskers (10-90%). Axenic treatments: light grey, non-axenic (control): dark grey; males: orange outline, females: purple outline. Details of statistical tests can be found in **Tables 1** and **2** . Drawing created using BioRender.

## Description

Interactions between members of the same species are mediated by many different factors, including the gut microbiome (Voung et al. *,* 2017). In addition to social behaviour, the gut microbiome modulates many other essential functions such as immunity and metabolism (Kim, 2018; Wong et al. *,* 2016). There are multiple routes from which the gut microbiome can influence host social behavior, including emission of chemical cues (Qiao et al., 2019) and modifying host physiology, specifically the gut-brain axis (Louwies et al., 2020) . Microbes within the gut hitchhike this neural highway and synthesize neurotransmitters and biologically active compounds that affect neural transmission of messages (Clarke et al., 2013) . There is also evidence suggesting the gut microbiome's involvement in reshaping brain structure and neural networks, which could have an impact on host social behaviour (Neufeld et al., 2011) .

Multiple studies have characterized the relationship between *D. melanogaster* and its microbiome in the context of social behaviour, which serves as foundational knowledge that can be used to understand the environmental mechanisms governing social space. For example, there is evidence supporting the involvement of bacterially-induced mating signals in the mediation of fly sexual preference. Indeed, flies prefer mates that exhibit similar microbial volatile profiles over those that are different (Sharon et al., 2010) ; those volatile profiles are dependent on microbial composition, which is itself influenced by the type of food substrate fed to the flies (Lizé et al., 2014; Sharon et al., 2010) . Furthermore, the egg-laying decision process of *D. melanogaster* females also involves the integration of chemical and pheromonal cues released by the microbiome of other mated females and foraging larvae (Durisko et al., 2014; Mansourian, 2016) .

Recently, a pro-social behaviour, sociability, has been reported to be affected by the microbiome, in a sex- and mating status-specific manner. Sociability is the measure of the preference flies have to occupy a shared space and engage in nonaggressive behaviour, in this case, on a food-patch (Scott et al. *,* 2018). Reduced microbiome in virgin males led them to be less social, but there was no effect on mated males nor on females regardless of their mating status (Panos et al., 2024) . As sociability and social space might be correlated (Yost et al., 2020) , we tested whether or not there would be an effect of the gut microbiome on social spacing in mated flies.

Social space is defined as the distance between an individual and its closest neighbour, and on average, the distance between *D. melanogaster* flies is approximately 1-2 body lengths (Simon et al., 2012) , an observation that has been reproduced multiple times and used in several recent studies (review: Brenman-Suttner et al., 2019; examples: Cao et al., 2022; Hope et al., 2019; Jia et al., 2022; Jiang et al., 2020; Kanellopoulos et al., 2020; Shilpa et al., 2021; Xie et al., 2018; Yost et al., 2020). We can assess the flies' preferred social space by quantifying several aspects of their distances to others. In this study, we report the distribution to the closest individual (which assesses only individual preferred distances), the number of flies within 4 body length (which assesses clustering), and the distribution of distance to all flies (which assesses the overall group size, Castells-Nobau et al., 2019; Jiang et al., 2020).

We tested the importance of the microbiome on social spacing by applying axenic treatments on three different *Drosophila* strains: Canton-S, Elwood, and BJS. The commonly used fly strain Canton S (CS) has been under laboratory conditions for more than a century. In contrast, Elwood and BJS were introduced to lab conditions within the last 15 years (see **Table 3** ). In addition to their different lab history, these 3 lines were collected in different geographical regions of North America: Canton, in northwest Ohio, for Canton-S in 1916, Elwood in the neighborhood of Elwood in Huntington, Long Island, in New-York State in 2011, and BJS in London, in southwest Ontario, in Canada in 2007.

We found that CS axenic flies form tighter clusters than non-axenic CS flies ( **Figure 1A** ), with a reduced number of flies within 4 body lengths (4 BL, ~1 cm) without any change in overall group structure, as seen in the distribution of distance to the closest and all neighbours ( **Figure 1B,C** ).

The Elwood strain showed no microbiome effect on the social space of males or females at close distance ( **Figure 1C** ), but when looking at the overall distribution of distance to closest and all neighbours, axenic females were closer compared to the control group ( **Figure 1D** ). Of note, the non-axenic Elwood flies were a lot further apart, as previously reported (McNeil et al., 2015) .

The differential effect of microbiome on social space between the two strains might be attributed to how recently the strain was caught. Hence, we tested another recently wild-caught strain, BJS, and observed the same effect of the microbiome as for Elwood, with only axenic females being closer to their nearest neighbour than non-axenic, without a change to cluster size ( **Figure 1G -I)** .

We also tested BJS treated with antibiotics to eliminate any possible *Wolbachia* infection (Hoffmann, 1988) . The antibiotic medium was prepared with tetracycline-HCl at a concentration of 0.3mg/ml. The fly colony was treated for one generation followed by at least three generations without antibiotics before any behavioural testing. We saw no difference at short distances in axenic BJS that had been treated with antibiotics, compared to the same non-axenic flies ( **Figure 1J,K )** . However, in the distribution to all neighbours, the antibiotic-treated BJS axenic males were further apart than the non-axenic males, as seen in Canton-S males. And antibiotic-treated BJS axenic females were closer than non-axenic females, as seen in the untreated axenic BJS females ( **Figure 1L** ).

These findings indicate that the flies depend on the existence of a gut microbiome to modulate how they space themselves relative to others in a group, in a complex strain- and sex-specific manner. Groups of recently caught females, but not males, came closer, whereas in the older lab strain, the group size was unchanged in females and increased in males, which were further apart, although in both cases, the clusters contained fewer flies. When the recently caught flies were treated with antibiotics, we observed an intermediate effect, with only the groups of males being further apart. So contrarily to what was reported by Panos et al. (2024) in the sociability experiment, performed on food patches, we did observe an effect of the microbiome in mated flies on the social space measure of pro-social interactions, where no food is present.

Drosophila microbiome is influenced by many factors including, but not only: the flies' diet (Obadia et al., 2018) , selection in the lab (see reviews by Ludington and Ja, 2020, and Douglas, 2018), genetic background (Dobson et al., 2015) , selection on stress and longevity (Kristensen et al., 2024) , exposure to overlapping generations (Wesseltoft et al., 2024; Guilhot et al., 2023) , and presence of the endobacterium *Wolbachia* (Henry, 2024) . *Wolbachia* is transmitted only by females, causing cytoplasmic incompatibility where progeny dies if the father is infected, but the mother is not. It can increase female fecundity in some strains (Serbus, 2008) , and may provide a fitness advantage in males depending on the genetic background (Fry et al., 2004) . The effects of *Wolbachia* on fitness, behavior, and mating vary based on host genetics, sex, and age (Shropshire et al., 2021; Layton et al., 2019) . A male-specific cost was observed with a viral gut infection (Vale and Jardine, 2015) . Although we did not check for the presence or absence of *Wolbachia* in our flies, we propose that lab selection, antibiotic treatments as well as geographical origin has led to behavioural differences in the effect of lack of microbiomes in the strains we tested. Microbial volatile compounds associated with specific food substrates may modulate *Drosophila* social behaviour (Davis et al., 2013) , in a sex-specific manner. Integration of these cues would be beneficial for *D. melanogaster* flies searching for food and mates. In addition, social spacing in *D. melanogaster* also depends on genetic background (McNeil et al., 2015) . For this reason, it is likely that different *D. melanogaster* strains would display varying microbiome-mediated social space. Follow-up experiments will have to test these differences at the molecular level, including genotyping as well as neural transcriptomics, and proteomics of the microbiomes.

In conclusion, our data support the microbiome's involvement in social interaction, to establish a preferred social space, but this effect is mediated by the sex of the flies, the genotype and history of the strain.

## Methods

***Fly strain and maintenance***

The fly strains are listed in **Table 3** . All fly stocks and population cohorts were reared in an incubator at 50 % humidity, 25 °C, and 12:12 light:dark cycle in the insect suite of the Biotron at the University of Western Ontario. The flies were maintained on nutrient-rich Jazzmix ^TM^ medium.

***Rearing axenic flies***

The protocol for preparing axenic groups is adapted from Tang et al. (2019). Briefly, flies were allowed to oviposit for 12 hours on apple juice agar (100 mL fruit juice, 100 mL dH2O, 4 g type II agar). Groups of 50 eggs were collected on sterile nylon filters, surface-sterilized with 70 % ethanol for five minutes and rinse thrice with sterile PBS. The filters were then inverted onto a thin layer of sterile Jazzmix ^TM^ medium to dislodge them. A small square of the medium containing the eggs was then transferred to autoclaved vials containing Jazzmix ^TM^ media. The vials were plugged with tight-fitting cellulose acetate plugs and paper caps to prevent contamination. The flies were then reared under standard conditions (25 °C, 50% relative humidity and a 12:12 h light:dark cycle) until they eclosed.

***Control group***

Control flies that still retain their microbiome were established using the same overall process. However, the control eggs were washed with sterile PBS instead of 70 % ethanol for 5 minutes. Since axenic flies take longer to develop, eggs for the control group were collected from the same parents but two days later, to assure all flies were the same age for the social space assay.

***Spot-checks for Sterility***

Three days prior to the behavioural experiment day, 1-4 days old axenic and control flies were transferred to sterile Jazzmix ^TM^ vials over a flame. Three flies were opportunistically selected from each axenic vial and homogenized in 200 μL of sterile PBS. A small aliquant of Jazzmix ^TM^ medium from each vial was also collected and homogenized in 200 μL of sterile PBS. A 10 μL aliquant from each sample was spot-plated on Yeast-Malt agar (YM) and incubated at 25 °C for 72 hours to assess microbial growth. Any vial that showed unexpected microbial growth were discarded.

Finally, to ensure axenic conditions were maintained throughout the assay, the flies in the chamber were euthanized by squirting 50 % ethanol into the chambers post-experiment. After one minute, three flies were opportunistically selected and thoroughly rinsed with sterile PBS to remove any ethanol residue. The flies were then homogenized in 200 μL of sterile PBS, 10 μL of each sample was spot-plated on YM agar and incubated at 25 °C for 72 hours to assess microbial growth. As a control, the process was repeated with control flies to ensure that the euthanizing process did not inadvertently kill the microbiome in the gut. Data from vials showing unexpected microbial growth were discarded. On average, we had a 90 % success rate in making axenic flies.

***Social Space Assay***

The behavioural assay was conducted as explained in McNeil et al. (2015), and adapted for axenic conditions. In short: 2 hours prior to an experiment, the axenic flies were separated by sex and sorted into autoclaved vials containing 15 individuals each. The flies were separated using a sterile spatula on an autoclaved acrylic pad close to a flame.

All the flies (axenic and non-axenic) were acclimated for at least 2 hours in the behavioural room (25 °C, 50 % humidity). They were then transferred into a vertical, two-dimensional-like triangular chamber. Once the flies settled, images were captured (at around 30 minutes) and processed using ImageJ to determine the different variables: number of flies within 4 body length, distance to closest neighbour, and distance to all neighbours (Image J routine available in (Yost et al., 2020) ). Each chamber contained 12 to 17 flies, and each treatment group had 9 independent biological replicates. There are a total of 4 treatment groups: control males, axenic males, control females, and axenic females. Each group was tested in triplicates every week, spanning three different weeks.

***Statistical Analysis***

Statistical analyses were conducted using GraphPad Prism 10 (GraphPad Software, La Jolla California USA, www.graphpad.com). We first performed ROUT analyses to remove outliers that would represent technical problems with reproducibility, before pooling the different biological replicates. We then assessed the normality of our data. The average number of flies within 4 body lengths (4BL) followed a normal distribution across the 9 independent repeats, so they were analysed using One-way-ANOVA, followed by a Sidak *post-hoc* multiple comparison analysis ( **Table 1).** The distributions of distance to closest neighbour or to all neighbours did not follow a normal distribution and were analyzed using a Kruskal-Wallis test, followed by a Dunn's *post-hoc* multiple comparison analysis ( **Table 2** ). Bolded *p* -values are considered significant.

&nbsp;

&nbsp;

&nbsp;

&nbsp;

&nbsp;

&nbsp;

**Table 1: One-way ANOVA table**

**Table:** 

**Strain**	**Panel**	***Df***	***F***	***p***	***Sidak post hoc***		
					**Comparison Group**	**Compared with**	***p***
Cs	A	(3, 28)	10	0.0001	Control Male **s**	**Axenic Males**	**0.0005**
					**Control Females**	**Axenic Females**	**0.0065**
Elwood	D	(3, 27)	2.1	0.1305	Control Males	Axenic Males	0.2487
					Control Females	Axenic Females	0.3995
BJS	G	(3, 40)	3.0	0.0438	Control Males	Axenic Males	0.9857
					Control Females	Axenic Females	0.0603
BJS antibiotics	J	(3, 32)	1.1	0.3818	Control Males	Axenic Males	0.1777
					Control Females	Axenic Females	0.9102
**One-Way ANOVA table** of number of flies within 4 body length (4BL). Two-tailed. Statistically significant results are bolded.							

&nbsp;

&nbsp;

&nbsp;

**Table 2: Kruskall Wallis table**

**Table:** 

**Strain / Experiment**		**Panel**	**Df**	**Kruskall Wallis** **Statistic (H)**	***p***	***Dunn's post hoc***		
						**Comparison group**	**Compared with**	***p***
Cs	CN	B	(4, 510)	2.4	0.5022	Control Males	Axenic Males	0.9422
						Control Females	Axenic Females	>0.9999
	AN	C	(4, 3380)	26.03	<0.0001	**Control Males**	**Axenic Males**	**0.0013**
						Control Females	Axenic Females	>0.9999
Elwood	CN	E	(4, 515)	5.6	0.132	Control Males	Axenic Males	>0.9999
						**Control Females**	**Axenic Females**	**0.0407**
	AN	F	(4, 3755)	30.41	<0.0001	Control Males	Axenic Males	0.1644
						**Control Females**	**Axenic Females**	**<0.0001**
BJS	CN	H	(4, 616)	17	0.0006	Control Males	Axenic Males	>0.9999
						**Control Females**	**Axenic Females**	**0.0138**
	AN	I	(4, 2014)	103	<0.0001	Control Males	Axenic Males	0.7655
						**Control Females**	**Axenic Females**	**<0.0001**
BJS antibiotics	CN	K	(4, 549)	6.4	0.0948	Control Males	Axenic Males	0.1154
						Control Females	Axenic Females	0.2666
	AN	L	(4, 4806)	106.0	<0.0001	**Control Males**	**Axenic Males**	**<0.0001**
						**Control Females**	**Axenic Females**	**<0.0001**
**Kruskall Wallis table.** Two-tailed. Statistically significant results are bolded. CN: distance to closest neighbour, AN: distance to all neighbours								

## Reagents

**Table 3: fly strains and reagents**

**Table:** 

**STRAIN**	**History**	**AVAILABLE FROM**
Canton-S	Isolated from Canton, a city in Northwest Ohio, USA in 1916 by C.B. Bridges (Stern, 1943) Simon lab stock (from Benzer's lab Caltech, 1998)	Simon lab stock and Bloomington stock Center # 64349
Elwood	Recently wild-caught strains: isolated from Elwood, a place in the Town of Huntington, on Long Island, in New York, USA in 2011 (McNeil et al. *,* 2015)	Simon lab stock
BJS	Isolated from London in Ontario, Canada in 2007 by B.J. Sinclair (Marshall & Sinclair, 2010) .	Simon and Sinclair lab stocks
**Reagents**	**DESCRIPTION**	**AVAILABLE FROM**
Jazzmix ^TM^	Drosophila food mixture (brown sugar, corn meal, yeast, agar, benzoic acid, methyl paraben and propionic acid) ready to use: simply add water.	Catalog No.AS153, Fisher Scientific
Phosphate buffered saline (PBS)	Prepared as per manufacturer's instructions	Catalog No. P4417, Sigma-Aldrich
Tetracycline-HCl	Antibiotic treatment	Catalog No. T7660, Sigma-Aldrich
YM agar	1% w/v glucose 0.5% peptone 0.3% malt extract 0.3% yeast extract 2% agar	Catalog No. 600-350-CG, Wisent Inc Catalog No. 800-157-LG, Wisent Inc Catalog No. CA90000-150, WR International Catalog No. 800-150-LG, Wisent Inc Catalog No. 820-010-CG, Wisent Inc
